# Supplementary material for: Effects of childhood trauma on mental health outcomes, suicide risk factors and stress appraisals in adulthood
Source: PLoS One. 2025 Jun 23;20(6):e0326120. doi: 10.1371/journal.pone.0326120 (PMC12185029; doi:10.1371/journal.pone.0326120)
Supplement: S1 Fig — All diagrams represent unstandardised B. (DOCX) [file pone.0326120.s001.docx]

**Supplementary Figure 1:** Path diagrams showing the non-significant moderation effects on outcome variables through social support, subjective SES, and suicide-related history. All diagrams represent unstandardised B.

**Moderator: Social Support (MSPSS)**

**Stress Appraisals**

**Stress-**

**Related**

**Outcomes**

b=-0.001, p=0.71

**Perceived Stress**

b=-0.01, p=0.23

**Defeat**

**Defeat-**

**Entrapment**

**Outcomes**

b=-0.05, p=0.22

**Entrapment**

b=-0.03, p=0.55

**Depression**

**Mental-**

**Health**

**Outcomes**

b=-0.04, p=0.08

**Anxiety**

b=-0.02, p=0.20

**Stress Appraisals**

**Stress-**

**Related**

**Outcomes**

b=-0.003, p=0.22

**Moderator: Subjective SES**

**Perceived Stress**

b=-0.007, p=0.40

**Defeat**

**Defeat-**

**Entrapment**

**Outcomes**

b=-0.022, p=0.70

**Entrapment**

b=-0.05, p=0.15

**Depression**

**Mental-**

**Health**

**Outcomes**

b=-0.012, p=0.36

**Anxiety**

b=-0.007, p=0.56

**Stress Appraisals**

**Stress-**

**Related**

**Outcomes**

b<-0.001, p=0.62

**Moderator: Suicide-related History**

**Perceived Stress**

b=0.01, p=0.80

**Anxiety**

b=-0.01, p=0.80

**Depression**

**Mental-**

**Health**

**Outcomes**

b=0.04, p=0.47

**Entrapment**

b=-0.49, p=0.72

**Defeat**

**Defeat-**

**Entrapment**

**Outcomes**

b=0.002, p=0.99
