# Supplementary material for: Effects of childhood trauma on mental health outcomes, suicide risk factors and stress appraisals in adulthood
Source: PLoS One. 2025 Jun 23;20(6):e0326120. doi: 10.1371/journal.pone.0326120 (PMC12185029; doi:10.1371/journal.pone.0326120)
Supplement: Attachment — Submitted filename: Reviewer.docx [file pone.0326120.s002.docx]

### ****Reviewer: Dr.Kwabena Acheampong****

### ****Effects of childhood trauma on mental health outcomes, suicide risk factors and stress appraisals in adulthood****

### ****Abstract****

### ****Lines 33–37****

**"The current study sought to: (1) investigate whether childhood trauma was associated to mental health outcomes (depression, anxiety), suicide risk factors (defeat, entrapment), and stress related outcomes (stress appraisal, perceived stress) in adulthood and (2) explore the indirect effects of childhood trauma on mental health outcomes and suicide risk factors through stress appraisal and perceived stress."**
Clearly defines two aims.
Suggested revision for conciseness and grammar:

"...associated **with** mental health outcomes..." (Correct "associated to" → "associated with")

### ****Line 38–41****

**"273 participants were recruited to online prospective study that consist of two sessions with one week interval. In session 1, the childhood trauma questionnaire was completed. In session 2, participants completed measures the level of depression, anxiety, defeat, entrapment, stress appraisal and perceived stress over the preceding week."**

Needs grammatical fixes:

"to **an** online prospective study"

"that **consisted** of two sessions"

"participants completed measures **assessing** the level of..."

Introduction

Summary of Strengths

- Well-organized and thorough.
- Cites foundational and recent literature.
- Clearly identifies research gaps.
- Introduces relevant theories (IMV model, transactional model).
- Logical flow from general to specific.

**Areas for Improvement**

Line	Issue	Suggestion

94 Wordiness	Reword to improve clarity: "how individuals appraise daily stressors"

101 Diction Replace "quotidian" with "everyday" unless required by journal style

136 Grammar	"remain" → "remained"

140–146 Readability	Consider using bullet points or shorter sentences to list the study aims

METHOD

Strengths Summary

- Clear structure and well-defined procedure.
- Ethical and transparent throughout.
- Inclusion/exclusion criteria and compensation are well explained.
- Pre-registration and REC approval enhance scientific credibility.

Suggestions Summary

Line	Issue	Suggestion

149	Formality	Replace "&" with “and” in subheading.

153	Clarity	Consider simplifying "lifetime history of suicide-related	behaviours".

Discussion

Overall Strengths

- Well-organized and comprehensive.
- Strong alignment with theoretical frameworks (e.g., IMV model, transactional stress model).
- Integrates prior studies effectively.
- Addresses both direct and indirect effects with nuance.

Areas for Improvement

Line	Issue	Suggested Revision

340	Placement of statistical control variables	Move “controlling for age and sex” to methods/results

347	Grammar	“Findings from current study are consistent...”

351	Phrasing	“associated to” → “associated with”

357	Awkward phrasing	“Worthy noticing” → “worth noting”

364	Diction	“happened in early life” → “experienced in early life”

375	Typo	“stres” → “stress”

386	Grammar	“shown” → “showed”

394	Verb form	“investigate” → “investigating”

396	Subject-verb agreement	“have long term effect” → “has long-term effects”

Implications

Overall Strengths

- Effectively links findings to real-world clinical applications.
- Clearly grounded in evidence-based therapy, especially CBT.
- Highlights mechanisms (stress appraisal) that can be targeted in intervention.

Suggested Revisions Summary

Line	Issue	Suggested Fix

398	Redundancy	“may have implications for potential interventions” → simplify

399–400 Grammar	“target at stress appraisals” → “targeting stress appraisals”

403	Awkward phrasing	“life events' outcomes” → “outcomes of life events”

405	Vague reference	Specify “this” more clearly

410	Citation clarity	Integrate citation into sentence more smoothly

413	Grammar	“core of CBT on” → “core focus of CBT is on”

Overall Strengths

Clear acknowledgment of sample and design limitations.

Well-structured recommendations for future research.

Highlights mediating mechanisms and theoretical contributions.

Suggestions are realistic, thoughtful, and grounded in current trends (e.g., diary methods, thematic analysis).

Suggested Revisions Summary

Line	Issue	Suggested Fix

420	Missing article	“current sample” → “the current sample”

421	Clarity	Rephrase to clarify screening and inclusion

429	Typo	Remove duplicate “the”

437	Grammar/clarity	“information on undergoing treatment” → “participants’ treatment status”

445	Repetition	Combine/simplify stress appraisal discussion

Overall Remarks

This article provides a thoughtful and empirically grounded exploration of the long-term psychological consequences of childhood trauma, with a particular focus on how stress appraisal and perceived stress mediate its effects on mental health and suicide risk factors. The study is timely, well-structured, and contributes meaningfully to the growing body of literature examining the cognitive and affective mechanisms linking early adversity to adult psychopathology.

The use of a two-session prospective design, inclusion of validated psychometric tools, and attention to both direct and indirect pathways of influence (e.g., via perceived stress and stress appraisals) are notable strengths. The study’s grounding in theoretical frameworks such as the Integrated Motivational-Volitional (IMV) model further strengthens its relevance and interpretative depth.

However, several methodological limitations reduce the generalizability of the findings. Notably, the lack of clinical screening for participants introduces the risk of confounding due to varying mental health statuses. Moreover, the cross-sectional nature of the stress appraisal measurement limits causal inferences, though the authors commendably suggest future longitudinal and ecological designs.

The discussion and implications are particularly strong, connecting empirical results to clinical intervention strategies, especially in the context of cognitive-behavioral therapy (CBT). The emphasis on refining CBT to better address maladaptive stress appraisals presents a promising avenue for intervention development.

The writing is generally clear, though some grammatical errors and awkward phrasings (e.g., repetition, article use, and sentence clarity) slightly detract from the professional tone. These could be easily corrected with careful proofreading.
