# Supplementary material for: Effects of childhood trauma on mental health outcomes, suicide risk factors and stress appraisals in adulthood
Source: PLoS One. 2025 Jun 23;20(6):e0326120. doi: 10.1371/journal.pone.0326120 (PMC12185029; doi:10.1371/journal.pone.0326120)
Supplement: Attachment — Submitted filename: Response to Reviewer.docx [file pone.0326120.s004.docx]

Dear Dr Acheampong,

Thank you for the feedback on the above paper and for the opportunity to submit a revision for further consideration. We have responded to all of the suggestions and we feel that the manuscript is now substantially improved. We hope that you will agree that we have been able to satisfactorily address the comments or provide justification where an alternative approach is taken and that you feel the manuscript is suitable for publication in *PLOS ONE*. Of course, we are very happy to respond to any further comments. The details of how we have addressed the specific issues raised are below and highlighted in yellow in the manuscript.

PONE-D-25-11717

### Reviewer #1: The manuscript was clearly written and well presented. the analyses was rigorous and adequate and the results was well presented. The study provides robust insight into the impact of childhood trauma on mental outcomes, suicidal risks and stress in later life

### **RESPONSE: We thank the reviewer for this positive comments.**

### **Reviewer #2**

Question for the authors: What informed the choice of one week between session 1 and session 2?

RESPONSE: Thanks for your question. We adopted a 1-week prospective design because we wanted to ensure that our assessment of childhood trauma was separated in time from the outcomes variables to help ensure completion of this measure did not contaminate the completion of the other measures as well as allowing us to more robustly test our predictions. One week was chosen specifically because we wanted to reduce the length of time commitment involved in taking part in the study, as our previous experience has indicated that having 7 days between timepoints helps to reduce drop-out.

### ****Abstract****

### ****Lines 33–37****

**"The current study sought to: (1) investigate whether childhood trauma was associated to mental health outcomes (depression, anxiety), suicide risk factors (defeat, entrapment), and stress related outcomes (stress appraisal, perceived stress) in adulthood and (2) explore the indirect effects of childhood trauma on mental health outcomes and suicide risk factors through stress appraisal and perceived stress."**
Clearly defines two aims.
Suggested revision for conciseness and grammar:

"...associated **with** mental health outcomes..." (Correct "associated to" → "associated with")

RESPONSE: As suggested, we have revised the aim to improve conciseness and grammar. Please refer to p. 3.

### ****Line 38–41****

**"273 participants were recruited to online prospective study that consist of two sessions with one week interval. In session 1, the childhood trauma questionnaire was completed. In session 2, participants completed measures the level of depression, anxiety, defeat, entrapment, stress appraisal and perceived stress over the preceding week."**

Needs grammatical fixes:

"to **an** online prospective study"

"that **consisted** of two sessions"

"participants completed measures **assessing** the level of..."

RESPONSE: Thank you very much for pointing this out. We have corrected the grammatical errors mentioned above (see p. 3).

Introduction

Summary of Strengths

- Well-organized and thorough.
- Cites foundational and recent literature.
- Clearly identifies research gaps.
- Introduces relevant theories (IMV model, transactional model).
- Logical flow from general to specific.

RESPONSE: Thank you for your positive comments.

**Areas for Improvement**

Line	Issue	Suggestion

94 Wordiness	Reword to improve clarity: "how individuals appraise daily stressors"

101 Diction Replace "quotidian" with "everyday" unless required by journal style

136 Grammar	"remain" → "remained"

140–146 Readability	Consider using bullet points or shorter sentences to list the study aims

RESPONSE: Thank you very much for your suggestions. We have corrected the grammatical issues you identified, replaced “quotidian” with “everyday” in line 101, and rewritten lines 94 and 140–146 to improve clarity and conciseness. Please see p. 6 & 8.

METHOD

Strengths Summary

- Clear structure and well-defined procedure.
- Ethical and transparent throughout.
- Inclusion/exclusion criteria and compensation are well explained.
- Pre-registration and REC approval enhance scientific credibility.

RESPONSE：Thank you for your positive comments.

Suggestions Summary

Line	Issue	Suggestion

149	Formality	Replace "&" with “and” in subheading.

153	Clarity	Consider simplifying "lifetime history of suicide-related	behaviours".

RESPONSE: Thank you very much for highlighting this issue. We have now addressed the problem—please see p. 9.

Discussion

Overall Strengths

- Well-organized and comprehensive.
- Strong alignment with theoretical frameworks (e.g., IMV model, transactional stress model).
- Integrates prior studies effectively.
- Addresses both direct and indirect effects with nuance.

RESPONSE：Thank you for your positive comments.

Areas for Improvement

Line	Issue	Suggested Revision

340	Placement of statistical control variables	Move “controlling for age and sex” to methods/results

347	Grammar	“Findings from current study are consistent...”

351	Phrasing	“associated to” → “associated with”

357	Awkward phrasing	“Worthy noticing” → “worth noting”

364	Diction	“happened in early life” → “experienced in early life”

375	Typo	“stres” → “stress”

386	Grammar	“shown” → “showed”

394	Verb form	“investigate” → “investigating”

396	Subject-verb agreement	“have long term effect” → “has long-term effects”

RESPONSE: Thank you very much for highlighting theseareas for improvement. We have implemented all the suggested changes. Please see p. 21-24.

Implications

Overall Strengths

- Effectively links findings to real-world clinical applications.
- Clearly grounded in evidence-based therapy, especially CBT.
- Highlights mechanisms (stress appraisal) that can be targeted in intervention.

RESPONSE：Thank you for your positive comments.

Suggested Revisions Summary

Line	Issue	Suggested Fix

398	Redundancy	“may have implications for potential interventions” → simplify

399–400 Grammar	“target at stress appraisals” → “targeting stress appraisals”

403	Awkward phrasing	“life events' outcomes” → “outcomes of life events”

405	Vague reference	Specify “this” more clearly

410	Citation clarity	Integrate citation into sentence more smoothly

413	Grammar	“core of CBT on” → “core focus of CBT is on”

RESPONSE: Thank you for your helpful suggestions. We have corrected the grammatical errors and awkward phrasing you identified. The section on CBT (lines 412–416) has been rewritten to integrate the citations more smoothly, and the wording at lines 401 and 408 has been revised for greater clarity. Please refer to p. 24 for the detailed changes.

Overall Strengths

Clear acknowledgment of sample and design limitations.

Well-structured recommendations for future research.

Highlights mediating mechanisms and theoretical contributions.

Suggestions are realistic, thoughtful, and grounded in current trends (e.g., diary methods, thematic analysis).

RESPONSE：Thank you for your positive comments.

Suggested Revisions Summary

Line	Issue	Suggested Fix

420	Missing article	“current sample” → “the current sample”

421	Clarity	Rephrase to clarify screening and inclusion

429	Typo	Remove duplicate “the”

437	Grammar/clarity	“information on undergoing treatment” → “participants’ treatment status”

445	Repetition	Combine/simplify stress appraisal discussion

RESPONSE: Thank you very much for your suggestions. We have corrected the grammatical and spelling errors you noted, including missing articles and typographical mistakes. In addition, we have rephrased the discussion of limitations concerning the screening and inclusion criteria (lines 423–427) to improve clarity. Finally, we have revised the summary of the stress-appraisal findings (lines 444–447) to make it more concise and straightforward. Please see pp. 25–26.

Overall Remarks

This article provides a thoughtful and empirically grounded exploration of the long-term psychological consequences of childhood trauma, with a particular focus on how stress appraisal and perceived stress mediate its effects on mental health and suicide risk factors. The study is timely, well-structured, and contributes meaningfully to the growing body of literature examining the cognitive and affective mechanisms linking early adversity to adult psychopathology.

The use of a two-session prospective design, inclusion of validated psychometric tools, and attention to both direct and indirect pathways of influence (e.g., via perceived stress and stress appraisals) are notable strengths. The study’s grounding in theoretical frameworks such as the Integrated Motivational-Volitional (IMV) model further strengthens its relevance and interpretative depth.

RESPONSE: Thank you so much for your positive comments.

However, several methodological limitations reduce the generalizability of the findings. Notably, the lack of clinical screening for participants introduces the risk of confounding due to varying mental health statuses. Moreover, the cross-sectional nature of the stress appraisal measurement limits causal inferences, though the authors commendably suggest future longitudinal and ecological designs.

RESPONSE: Thank you for your thoughtful comments. As you have noted—and as we detail in the study’s limitations—this research does have certain constraints. These limitations are explicitly acknowledged and discussed in the Discussion section. We believe they do not materially weaken the findings and will instead contribute meaningfully to the existing literature and prompt further research in this area.

The discussion and implications are particularly strong, connecting empirical results to clinical intervention strategies, especially in the context of cognitive-behavioral therapy (CBT). The emphasis on refining CBT to better address maladaptive stress appraisals presents a promising avenue for intervention development.

RESPONSE: Thank you so much for your positive comments.

The writing is generally clear, though some grammatical errors and awkward phrasings (e.g., repetition, article use, and sentence clarity) slightly detract from the professional tone. These could be easily corrected with careful proofreading.

RESPONSE: Thank you very much for all your suggestions on grammar and phrasing. We have incorporated every comment, and have made additional edits throughout to improve the sentence clarify and grammar. We are confident that the revised manuscript is now substantially improved.
